# SH2 domain-containing phosphatase 1 regulates pyruvate kinase M2 in hepatocellular carcinoma

**DOI:** 10.18632/oncotarget.7923

**Published:** 2016-03-05

**Authors:** Wei-Tien Tai, Man-Hsin Hung, Pei-Yi Chu, Yao-Li Chen, Li-Ju Chen, Ming-Hsien Tsai, Min-Husan Chen, Chung-Wai Shiau, Yin-Pin Boo, Kuen-Feng Chen

**Affiliations:** ^1^ Department of Medical Research, National Taiwan University Hospital, Taipei, Taiwan; ^2^ National Center of Excellence for Clinical Trial and Research, National Taiwan University Hospital, Taipei, Taiwan; ^3^ Division of Meidcal Oncology, Department of Oncology, Taipei Veterans General Hospital, Taipei, Taiwan; ^4^ Program in Molecular Medicine, School of Life Science, National Yang-Ming University, Taipei, Taiwan; ^5^ Department of Pathology, Show Chwan Memorial Hospital, Changhua City, Taiwan; ^6^ School of Medicine, Fu Jen Catholic University, New Taipei City, Taiwan; ^7^ Department of Surgery, Changhua Christian Hospital, Changhua, Taiwan; ^8^ Institute of Biopharmaceutical Sciences, National Yang-Ming University, Taipei, Taiwan; ^9^ School of Medicine, National Yang-Ming University, Taipei, Taiwan; ^10^ School of Medicine, Kaohsiung Medical University, Kaohsiung, Taiwan

**Keywords:** PKM2, SHP-1, PTPN6, sorafenib, HCC

## Abstract

Pyruvate kinase M2 (PKM2) is known to promote tumourigenesis through dimer formation of p-PKM2^Y105^. Here, we investigated whether SH2-containing protein tyrosine phosphatase 1 (SHP-1) decreases p-PKM2^Y105^ expression and, thus, determines the sensitivity of sorafenib through inhibiting the nuclear-related function of PKM2. Immunoprecipitation and immunoblot confirmed the effect of SHP-1 on PKM2^Y105^ dephosphorylation. Lactate production was assayed in cells and tumor samples to determine whether sorafenib reversed the Warburg effect. Clinical hepatocellular carcinoma (HCC) tumor samples were assessed for PKM2 expression. SHP-1 directly dephosphorylated PKM2 at Y105 and further decreased the proliferative activity of PKM2; similar effects were found in sorafenib-treated HCC cells. PKM2 was also found to determine the sensitivity of targeted drugs, such as sorafenib, brivanib, and sunitinib, by SHP-1 activation. Significant sphere-forming activity was found in HCC cells stably expressing PKM2. Clinical findings suggest that PKM2 acts as a predicting factor of early recurrence in patients with HCC, particularly those without known risk factors (63.6%). SHP-1 dephosphorylates PKM2 at Y105 to inhibit nuclear function of PKM2 and determines the efficacy of targeted drugs. Targeting PKM2 by SHP-1 might provide new therapeutic insights for patients with HCC.

## INTRODUCTION

Abnormal glucose metabolism enhances biosynthesis and cell proliferation and is, therefore, a vital component of tumorigenesis. Unlike pyruvate kinase M1 (PKM1), which is dominant as it is constitutively active; pyruvate kinase M2 (PKM2) acts as a gatekeeper, maintaining aerobic glycolysis, also known as the Warburg effect, and is expressed in embryonic and cancer cells. [1, 2] PKM2 is predominantly expressed in cancer cells with low-PK activity, and has dimeric formation rather than the highly-active, tetrameric formation. [3, 4] The switch between tetramer and dimer formation is determined by the phosphorylation of PKM2^Y105^. [5, 6] Recently, PKM2 has been identified to have nonglycolytic activity in tumorigenesis. In particular, the nuclear location of PKM2 is associated with cell proliferation. In addition to its glycolytic function, PKM2 also acts as a protein kinase targeting histone H3 [7] and STAT3 [8] to promote tumorigenesis and gene transcription. On the other hand, several growth signals, such as EGF [7, 9-12] and FGF [5, 13], have been reported to enhance the nucleus-dependent proliferative activity of PKM2 by protein tyrosine phosphorylation. Also, PKM2 acts as a coactivator for hypoxia-inducible factor 1 [14]. Thus, here, we explored the hypothesis that the protein tyrosine phosphatase (PTP), which regulates the nucleus-dependent PKM2, contributes to the sensitivity of targeted drugs by reversing the nuclear location of PKM2.

Here, we explored the molecular mechanism by which SHP-1 affects the enzymatic and nucleus-dependent proliferative activity of PKM2. After disclosing the novel action of SHP-1 in PKM2, we tested the potential effect of SHP-1-mediated PKM2 dephosphorylation as therapeutic target of HCC and revealed that the sensitivity of targeted drugs can be determined by PKM2 *in vitro* and *in vivo*. Targeting PKM2 by SHP-1 activation may provide new therapeutic insights for the treatment of HCC.

## RESULTS

### SHP-1 dephosphorylates PKM2 at Y105

**Figure 1:**
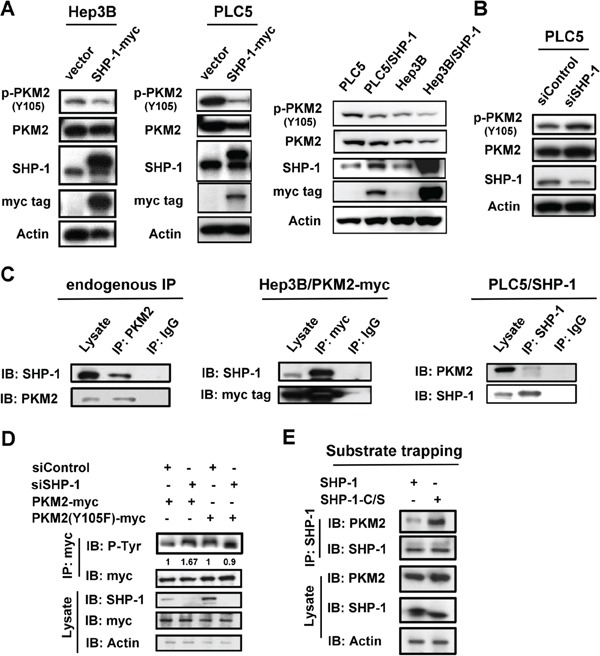
SHP-1 dephosphorylates PKM2 at Y105 **A.** *Left*, The transient effect of SHP-1 on p-PKM2^Y105^. PLC5 and Hep3B cells were transiently transfected with PKM2 for 48 h. *Right*, SHP-1 induces PKM2^Y105^ dephosphorylation in HCC cells stably expressing SHP-1. **B.** Silencing SHP-1(*PTPN6*) upregulates p-PKM2^Y105^. **C.** Identification of PKM2 as an interaction partner of SHP-1. The HCC cell lysate (1 mg) were used in immunoprecipitation. **D.** Silencing SHP-1 did not further affect the phospho-status of PKM2 in cells transfected with PKM2 Y105F mutant. PLC5 cells transfected with wild-type or mutant PKM2 and SHP-1(*PTPN6*) siRNA or control siRNA were analyzed by IP and IB as indicated. **E.** PKM2 is a phospho-substrate of SHP-1 as demonstrated by substrate trapping assay. The interaction affinity of wild-type or catalytic-dead SHP-1 (C453S) with PKM2 was assayed.

The phosphorylation of PKM2^Y105^ is known to play a critical role in the Warburg effect and cell proliferation. [6] First, we investigated the potential effect of SHP-1 on p-PKM2^Y105^. Both transient and stable expression of SHP-1 caused dephosphorylation of p-PKM2^Y105^ in HCC cells (Figure 1A). Conversely, the induction of p-PKM2^Y105^ was found in PLC5 cells with silenced SHP-1 (Figure 1B). Further, IP assay of both endogenous and overexpression showed that PKM2 interacts with SHP-1 in HCC cells (Figure 1C). To validate the specificity of SHP-1 to phospho-Y105 residue, we tested the phospho-status of PKM2 in PLC5 cells with silenced SHP-1. Silencing SHP-1 did not further enhance the level of p-PKM2 in PKM2-Y105F-expressing HCC cells when compared to wild-type PKM2, suggesting that SHP-1 dephosphorylates PKM2 at Y105 specifically (Figure 1D). We next assessed the affinity of PKM2 to the substrate trapping mutant of SHP-1 (SHP-1-C/S), in which the conserved C453 residue was mutated to generate an inactive enzyme that locks the substrate in its catalytic pocket. [15] This substrate trapping mutant of SHP-1 could bind more PKM2, indicating that PKM2 is a substrate of SHP-1 (Figure 1E). These results disclosed the potential regulation of p-PKM2^Y105^ by SHP-1 through dephosphorylation.

### Effect of SHP-1 on nucleus-dependent PKM2

**Figure 2:**
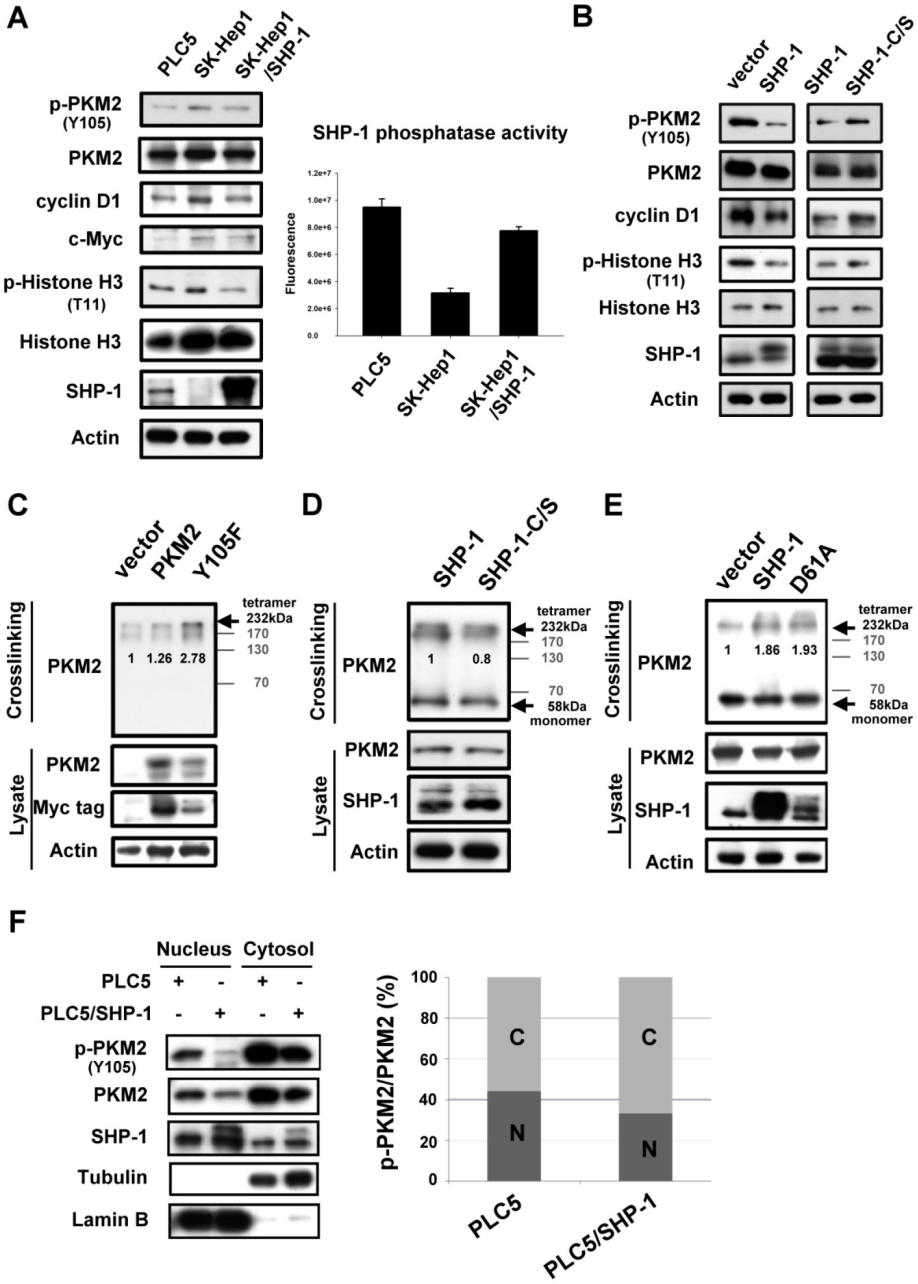
Effect of SHP-1 on nucleus-dependent PKM2 **A.** *Left*, P-PKM2-related oncogenic proteins were analyzed in PLC5, SK-Hep1, and SK-Hep1 stably expressing SHP-1. *Right*, SHP-1 phosphatase activity. *Columns*, mean; *bars*, SD (n = 3-6). **B.** P-PKM2-dependent oncogenic proteins in PLC5 cells with wild-type or catalytic-dead mutant (C/S). **C.** The Y105F mutant of PKM2 increases the formation of tetrameric PKM2. PLC5 cells with wild-type or mutant PKM2 were crosslinked by 1% glutaraldehyde before IB analysis. **D** and **E.** The effect of SHP-1 on tetrameric PKM2. The catalytic-dead (C/S) and constitutively active mutant (D61A) of SHP-1 reduced and increased the tetramer formation of PKM2 respectively. **F.** SHP-1 decreases the nuclear percentage of p-PKM2.

Dimer-dependent nuclear PKM2 is thought to mediate histone H3 phosphorylation and promote downstream oncogene transcription to induce tumorigenesis. [7] As phosphorylation of PKM2^Y105^ that disrupts formation of active, tetrameric PKM2 is potentially required for the nuclear location, [6] we next sought to elucidate whether SHP-1 affects the proliferative activity of PKM2 in the nucleus. First, HCC cells with different levels of expression of SHP-1 were used to test the p-PKM2-related oncogenesis. SK-Hep1 cells expressing higher levels of p-PKM2^Y105^ and lower levels of SHP-1, had a greater effect on p-histone H3-dependent oncogene expression, such as cyclin D1 and c-Myc, compared to PLC5 cells (Figure 2A, *Left*). In addition, SK-Hep1 stably expressing SHP-1 restored H3-dependent oncogene expression through dephosphorylation of PKM2^Y105^. The SHP-1 phosphatase activity also correlated with the phospho-status of PKM2^Y105^ in these HCC cell lines (Figure 2A, *Right*). To compare the effect of different SHP-1 activity on PKM2, we further investigated the wild-type and catalytic-dead (C/S) mutants in nuclear PKM2-related oncogene expression. In PLC5 cells, SHP-1-induced dephosphorylation of PKM2^Y105^ caused downregulation of p-Histone H3^T11^, and cyclin D1. Compared to wild-type SHP-1; catalytic-dead SHP-1 reversed the nuclear PKM2-mediated oncogenic expression (Figure 2B).

As previously described, PKM2 is found as active tetramers or less active dimmers. [3, 4] However, in tumor cells, PKM2 is predominantly in dimeric formation with low pyruvate kinase activity. To further investigate whether SHP-1-related dephosphorylation of PKM2^Y105^ affects the switch between dimer and tetramer formation, we next assayed the shifts of tetrameric PKM2 at different levels of SHP-1 activity. The dephosphorylated mutant of PKM2 (Y105F) increased the formation of tetrameric PKM2 (Figure 2C and Supplementary Figure 1), suggesting that p-PKM2^Y105^ indeed resulted in a shift from tetrameric formation to the dimeric formation which is strongly correlated to cell proliferation. The catalytic-dead SHP-1 (C/S) had a reduced percentage of tetrameric PKM2; however, constitutively active SHP-1 (D61A) showed increased tetramer formation of PKM2, indicating that SHP-1-mediated phospho-status of PKM2^Y105^ potentially alters the ratio of tetrameric-to-dimeric PKM2 (Figure 2D, 2E, and Supplementary Figure 2). The less active dimeric PKM2 is thought to mediate the nucleus-dependent proliferative activity that plays a major role in the nonglycolytic function of PKM2. [8] We found that the percentage of p-PKM2 in the nucleus was also decreased in PLC5 cells stably expressing SHP-1 (Figure 2F and Supplementary Figure 3). These findings thus demonstrate that SHP-1-mediated phospho-change of PKM2^Y105^ is required for the proliferative function of PKM2 in the nucleus.

### Sorafenib promotes the enzymatic function of PKM2 through dephosphorylation of PKM2^Y105^

**Figure 3:**
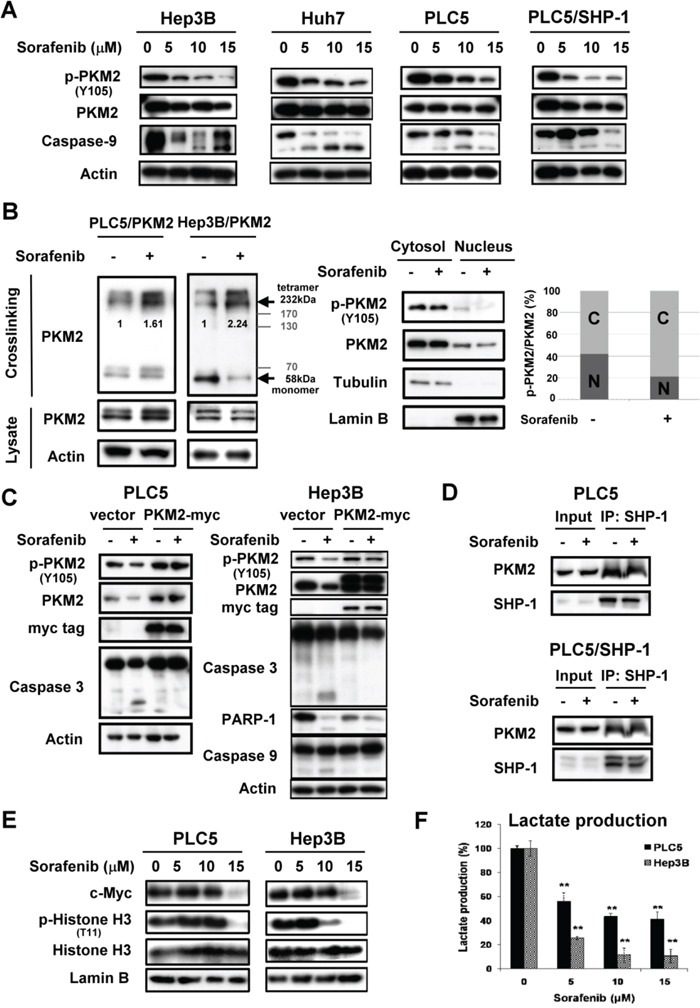
Sorafenib promotes enzymatic function of PKM2 through dephosphorylation of PKM2^Y105^ **A.** Sorafenib downregulates the p-PKM2^Y105^ at the indicated doses after treatment for 24 h. **B.** *Left*, Sorafenib increases the tetramer formation of PKM2 at a dose of 10 μM. *Right*, Sorafenib reduces the nuclear fractions of p-PKM2. **C.** Overexpression of PKM2 restores sorafenib-induced apoptosis in HCC cells. **D.** Sorafenib did not affect the interaction of SHP-1 and PKM2. **E.** Sorafenib downregulates the expression of PKM2-dependent oncogenic proteins. HCC cells were treated with sorafenib at the indicated doses for 24 h and the nuclear fractions were extracted for IB assay. **F.** Sorafenib significantly reduces the production of lactate at the indicated doses.

As SHP-1 was found to dephosphorylate PKM2^Y105^, we next investigated the therapeutic potential of SHP-1 in HCC treatment through this mechanism. Sorafenib, the first and only approved targeted drug for HCC, is reported to be a SHP-1 agonist. [16] Interestingly, sorafenib induced dephosphorylation of PKM2^Y105^ at apoptosis relevant doses in the HCC cell lines. Of note, sorafenib downregulated p-PKM2^Y105^ more effectively in PLC5 cells stably expressing SHP-1 compared to parental PLC5 cells (Figure 3A). This p-PKM2^Y105^ downregulation correlated with increased tetramer formation of PKM2 (Figure 3B, *Left* and Supplementary Figure 4) and decreased the levels of nuclear PKM2 under sorafenib treatment (Figure 3B, *Right* and Supplementary Figure 5). This finding, that sorafenib downregulates the phospho-status of PKM2^Y105^ and promotes active tetrameric PKM2 formation, may represent the contribution of PKM2 to the drug effect of sorafenib.

Further, in PKM2-overexpressing PLC5 and Hep3B cells, we failed to find active apoptotic markers, such as caspase-3 and PARP-1, compared to parental cells, suggesting that PKM2 plays a role in the effect of sorafenib on HCC cell death (Figure 3C). However, the interaction status of SHP-1 and PKM2 was not affected by sorafenib (Figure 3D). To elucidate whether sorafenib-induced dephosphorylation of PKM2^Y105^ leads to inhibition of the nucleus-dependent proliferative activity, we further analyzed the nuclear extract upon sorafenib treatment. Consistent with the results of SHP-1 on PKM2, sorafenib also induced downregulation of p-Histone H3^T11^ and c-Myc in a dose-dependent manner (Figure 3E). Also, lactate production was significantly inhibited by sorafenib treatment, indicating that decreased level of p-PKM2^Y105^ indeed resulted in the promotion of PKM2 enzymatic function and the opposition of the Warburg effect (Figure 3F).

### PKM2 determines the efficacy of targeted drugs in HCC cell lines

To explore the therapeutic potential of PKM2 for HCC treatment, we tested whether PKM2 affects the sensitivity of targeted drugs via SHP-1. Sorafenib, acting as an anti-VEGFR tyrosine kinase inhibitor (TKI), is the first and only approved targeted drug for the treatment of HCC [17-21]; however, in recent phase III trials, other anti-VEGFR TKIs, such as brivanib and sunitinib, did not show clinical benefit. The precise drug target that determines the efficacy for these TKIs is still unknown.

**Figure 4:**
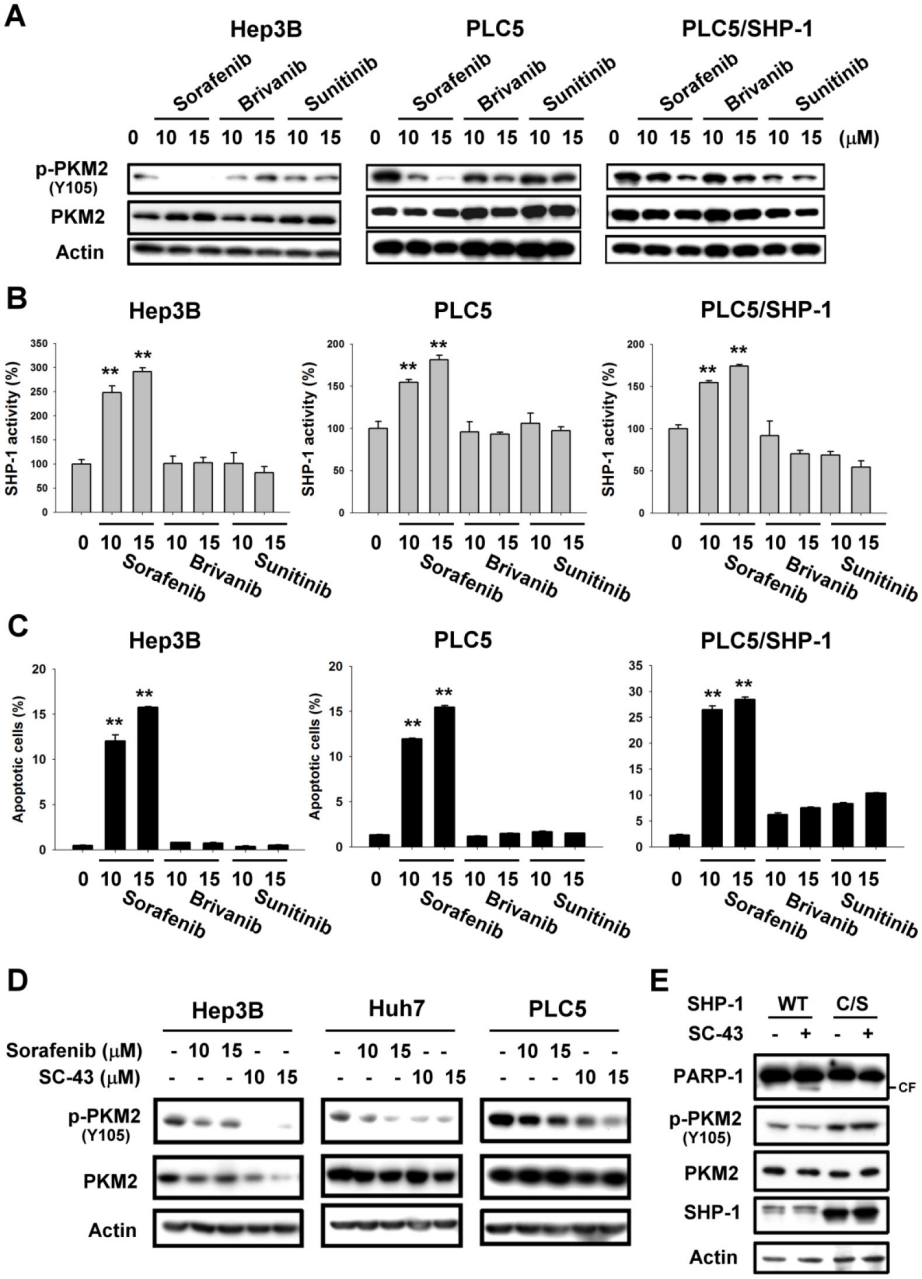
PKM2 determines the efficacy of targeted drugs in HCC cell lines **A.** Sorafenib, but not brivanib or sunitinib, downregulates the p-PKM2^Y105^ at the indicated doses after treatment for 24 h. **B.** Sorafenib, but not brivanib or sunitinib, elevates SHP-1 activity. **C.** Apoptosis in sorafenib, brivanib, and sunitinib-treated HCC cells. *Columns*, mean; *bars*, SD (n ≥ 3).*, *P* < 0.05, **, *P* < 0.01. **D.** Potent SHP-1 agonist, SC-43, downregulates more p-PKM2^Y105^ than sorafenib. **E.** Catalytic-dead SHP-1 (C/S) reverses SHP-1 agonist-induced p-PKM2^Y105^ downregulation and apoptosis.

Brivanib and sunitinib did not have a significant effect on PKM2^Y105^ dephosphorylation in PLC5 and Hep3B cells (Figure 4A). But in SHP-1-expressing PLC5 cells, brivanib and sunitinib exhibited the same ability to downregulate p-PKM2^Y105^ as sorafenib. Further, only sorafenib was able to elevate SHP-1 phosphatase activity and induce effective apoptosis in HCC cells, in comparison with brivanib and sunitinib, suggesting that p-PKM2^Y105^ downregulation may determine the sensitivity of these targeted drugs via SHP-1 activation (Figure 4B and 4C). We, therefore, next applied a more potent SHP-1 agonist, SC-43, [16] derived from sorafenib to investigate the effect on p-PKM2^Y105^ downregulation. A more potent effect on p-PKM2^Y105^ downregulation was shown in HCC cells with SC-43 treatment compared to sorafenib (Figure 4D). The catalytic-dead mutant of SHP-1 counteracted the effect of SC-43 on p-PKM2^Y105^ and apoptosis (Figure 4E). These data strongly suggest that PKM2 plays a critical role in targeted drug-induced apoptosis by SHP-1.

### *In vivo* effect of PKM2 on sorafenib-treated HCC xenograft

**Figure 5:**
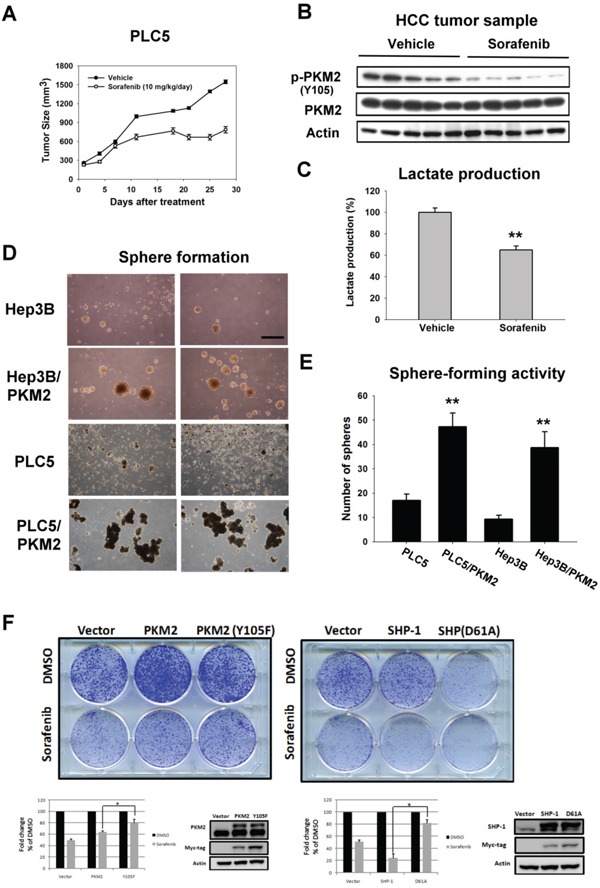
In vivo effect of PKM2 on sorafenib-treated HCC xenograft **A.** Tumor growth inhibition by sorafenib in PLC5-bearing mice. Mice received sorafenib at 10 mg/kg/day (p.o.) and tumor growth was measured twice weekly. *Points*, mean; *bars*, SD (n = 8). **B.** Analysis of p-PKM2^Y105^ and PKM2 in PLC5 tumors. **C.** Sorafenib significantly reduced the lactate production in the tumor sample. *Column*, mean; *bars*, SD (n = 8). **D.** Sphere formation. HCC cells transfected with vector or PKM2 were seeded in an ultra-low attachment 6-well plate for 14 days. **E.** Quantitative data of sphere formation. PKM2 increases the sphere formation in HCC cells. **F.** Colony-forming activity. PKM2 (Y105F) and SHP-1 (D61A) decreased the anti-proliferation effect of sorafenib.

To assess the therapeutic potential of PKM2 in a clinically relevant model, we tested the phospho-status of PKM2^Y105^ on HCC xenograft with sorafenib treatment. As previously described, [22] sorafenib at a dosage of 10 mg/kg/day exhibits almost 50 % inhibitory activity in PLC5-bearing mice (Figure 5A). To further validate the effect of PKM2 in sorafenib-treated tumor extract, we collected the tumor sample at the end of treatment. Interestingly, dephosphorylation of PKM2^Y105^ and inhibition of lactate production were seen in sorafenib-treated tumor lysate reflecting that seen in the *in vitro* system indicating that sorafenib also affects the enzymatic activity of PKM2 to reverse the Warburg effect *in vivo* (Figure 5B and 5C).

To confirm the proliferative activity of PKM2 in HCC, we checked the tumor-forming ability by sphere formation (Figure 5D). We found more spheres in HCC cells stably expressing PKM2 in comparison with parental cells (Figure 5E). Moreover, the colony-forming activity showed that PKM2 (Y105F) and SHP-1 (D61A) decreased the anti-proliferation effect of sorafenib, suggesting that PKM2 and SHP-1 could be therapeutic determinants of sorafenib in HCC cells (Figure 5F). Taken together, these results suggest that PKM2 may function as an oncogene in tumorigenesis and provide new insights into therapeutic strategies for HCC.

### PKM2 as a biomarker of early recurrence in HCC patients

**Figure 6:**
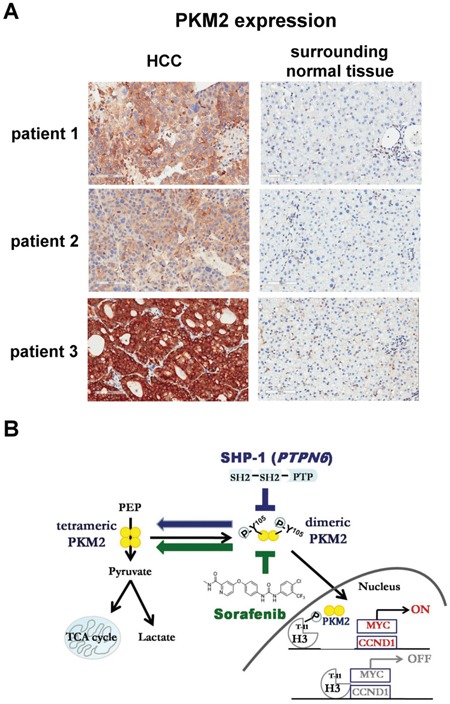
Level of PKM2 expression in clinical HCC samples **A.** The representative immunohistochemical patterns of PKM2 in clinical HCC and surrounding normal tissues. **B.** Summary model. SHP-1 dephosphorylates p-PKM2^Y105^ and further affects the nucleus-related cell proliferation. The SHP-1-dependent PKM2^Y105^ dephosphorylation also determines the sensitivity of sorafenib in HCC.

Furthermore, we studied the presence of PKM2 in clinical tumor samples obtained from 147 HCC patients who received curative surgical resection and 59 patients (40.1%) had positive PKM2 staining revealed by IHC (Supplementary Table 1). Within our cohort, 17 patients developed disease recurrence of tumor within half year. It is worth of note that 9 patients (52.9%) within this early-relapsed cohort had positive PKM2 expression. Furthermore, if we exclude patients with known risk factors for early relapse, namely large and/or multiple tumors, positive nodal involvements, and major vascular invasion, risk of early recurrence in PKM2-expressed patients was significantly higher than PKM2-negative patients (87.5% v.s. 12.5%; *p* < 0.001). Our data suggest the potential role of PKM2 in predicting early recurrence in patients with HCC, particularly those without known risk factors. The representative immunohistochemical patterns of PKM2 in clinical HCC tissues were showed (Figure 6A). Our data suggest that PKM2 may have a potential role in predicting early recurrence in patients with HCC, particularly those without known risk factors.

## DISCUSSION

PKM2 is expressed in tumor cells and plays an important role in the shift to aerobic glycolysis associated with tumor progression. [3, 23] Loss of PKM2 is known to extend disease latency of myeloid leukemia in mice. [24] In some solid tumors such as esophageal squamous carcinoma, [25] colorectal cancer, [26] and breast cancer, [27] the expression of PKM2 also accompanies aggressive tumor progression. In addition to cancer models, hedgehog signaling was reported to activate the Warburg effect to control the fate of hepatic stellate cells. [28] Our clinical data suggest that PKM2 has a potential role in predicting early recurrence in patients with HCC, particularly those without known risk factors (63.6%), implying that PKM2 may be a relevant tumor marker and therapeutic target for HCC treatment.

However, the underlying molecular mechanism by which PKM2 affects tumorigenesis is still poorly understood. To date, evidence suggests that post-translational modification (PTM) of PKM2 mediates oncogenesis in several tissue types. The phosphorylation of PKM2^T454^ by PIM2 led to an increase in PKM2 expression and the Warburg effect in cancer cells. [29] Phosphorylated-PKM2^S37^ through EGFR-activated ERK2 promotes nuclear translocation of PKM2, acting as a coactivator of β-catenin in human glioblastoma cells. [10] The acetylation of PKM2^K305^ decreases the enzymatic activity of PKM2 and promotes its degradation by association with HSC70 that acts as a chaperone for chaperone-mediated autophagy. [30] The oxidation of PKM2 at Cys358 caused antioxidant responses to obtain survival advantages under oxidative stress. [31] In addition, the phosphorylation of PKM2^Y105^ by FGFR1 switches the formation of active, tetrameric PKM2 to less active, dimeric PKM2, and confers a metabolic advantage towards tumor proliferation. [6] Collectively, these reports disclose the importance of PKM2, especially the nucleus-dependent PKM2, in oncogenesis. According to our finding (Figure 2), SHP-1-mediated PKM2^Y105^ dephosphorylation contributed to a shift towards more active, tetrameric PKM2 and resulted in reduced nuclear location downregulating oncogene expression, such as c-Myc and cyclin D1. Moreover, constitutively active SHP-1 (D61A) had a potent effect on the percentage of tetrameric PKM2 in the same way as phospho-mutant of PKM2 (Y105F), indicating that SHP-1 determines the shift in dimeric/tetramer PKM2 and subsequent nucleus location via PKM2^Y105^ dephosphorylation. Notably, PKM2 (Y105F) confirmed the specificity of SHP-1 to p-PKM2^Y105^ when SHP-1(*PTPN6*) siRNA was applied (Figure 1D).

SHP-1(*PTPN6*) was first identified in hematopoietic cells and is implicated in various hematopoietic signaling processes, such as integration of immunoreceptor tyrosine-based activation motif (ITAM)-mediated inhibitory signal, [32] B-cell development and function, [33] and anti-tumor function of natural killer (NK) cells. [34] However, the role of SHP-1 in solid tumors is still not very clear. Given that SHP-1 dephosphorylates PKM2^Y105^ reversing its nucleus-dependent proliferative activity, the discovery of small molecules targeting SHP-1 may be a promising strategy through which to inhibit PKM2-related nonglycolytic function and the Warburg effect for cancer treatment.

Several studies have shown the therapeutic potential of PKM2 for cancer treatment. For example, mRNA level of PKM2 acts as an independent predictive biomarker of poor outcome in advanced NSCLC patients with platinum-based chemotherapy. [35] The natural activators of PKM2, SAICAR [36] and serine [37], have been identified to promote the pyruvate kinase activity of PKM2. In addition, the discovery of PKM2 activators, like TEPP-46 and DASA-58 [38] also bind PKM2 directly to cause a constitutively active enzyme state that interferes with anabolic metabolism. The combinational use of PKM2 activators and SHP-1 agonists may be a promising approach to reverse PKM2-mediated Warburg effect for therapeutic purposes.

In conclusion, in this study we demonstrated that SHP-1 dephosphorylates PKM2^Y105^ to inhibit the Warburg effect and nucleus-dependent cell proliferation, and the dephosphorylation of PKM2^Y105^ by SHP-1 determines the efficacy of targeted drugs for HCC treatment (Figure 6B).

## MATERIALS AND METHODS

### Cell culture

The Huh-7 HCC cell line was obtained from the Health Science Research Resources Bank (HSRRB, Osaka, Japan; JCRB0403). PLC/PRF/5 (PLC5) and Hep3B were obtained from American Type Culture Collection (Manassas, VA). All cells obtained from HSRRB or ATCC were immediately expanded and frozen down such that all cell lines could be restarted every 3 months from a frozen vial of the same batch of cells. No further authentication was conducted in our laboratory.

### Reagents

Sorafenib (Nexavar) and brivanib (BMS-582664) were kindly provided by Bayer Pharmaceuticals and Bristol-Myers Squibb respectively. Sunitinib (Sutent) was obtained from Pfizer. For cell-based studies, targeted drugs at various concentrations were dissolved in DMSO and then added to the cells in serum-free DMEM medium. Glutaraldehyde used as a crosslinker, was from Sigma-Aldrich (St. Louis, MO).

### Antibodies

Antibodies for immunoblotting such as cyclin D1, actin, tubulin, and PARP-1 were purchased from Santa Cruz Biotechnology (San Diego, CA). Other antibodies such as p-PKM2^Y105^, PKM2, p-Histone H3^T11^, Histone H3, and myc-tag were from Cell Signaling (Danvers, MA). SHP-1 antibody was purchased from Abcam (Cambridge, MA).

### Plasmid, siRNA and transfection

Plasmids encoding the human PKM2 and SHP-1 mutants were cloned into pCMV6-Entry vector with myc-tag. All the truncated mutants were confirmed by DNA sequence and assayed for their expression level in HCC cells. Catalytic dead mutant of SHP-1 at Cys 453 replaced with Ser retains its ability to bind phospho-tyrosine but competes with endogenous SHP-1 enzyme activity. Plasmid for pJ3-SHP-1 (C453S) was purchased from Addgene plasmid repository (http://www.addgene.org/). Smart-pool siRNA, including control (D-001810-10), SHP-1 (PTPN6, L-009778-00-0005) were all purchased from Dharmacon (Chicago, IL). For transient expression, plasmids or siRNA were pre-transfected with lipofetamine 2000 (Invitrogen, CA) for 24 h and then processed with indicated treatment for another 24 h. The procedure has been described previously [39, 40].

### Apoptosis analysis

The apoptotic percentage was analyzed by sub-G1 demonstrated in flow cytometry. Sorafenib, brivanib or sunitinib-treated cells were fixed in 70% ethanol at −20°C overnight and then stained with 20 μg/ml propidium iodide (PI). The protocol for flow cytometry follows the manufacturer's suggestion [41].

### SHP-1 phosphatase activity

After treatment with sorafenib, brivanib, or sunitinib, HCC protein extract was incubated with anti-SHP-1 antibody in immunoprecipitation buffer (20 mM Tris-HCl (pH 7.5), 150 mM NaCl, 1 mM EDTA, 1% NP-40, and 1% sodium deoxycholate) overnight. Protein G-Sepharose 4 Fast flow (GE Healthcare Bio-Science, NJ) was added to each sample, followed by incubation for 3 h at 4°C with rotation. A RediPlate 96 EnzChek Tyrosine Phosphatase Assay Kit (R-22067) was used for SHP-1 activity assay (Molecular Probes, Invitrogen, CA).

### Sphere formation

PLC5 or Hep3B cells stably expressing PKM2 were plated in ultra-low attachment 6-well plates. After two weeks, the sphere was imaged and sphere formation was quantified.

### Animal study

Male NCr athymic nude mice (5-7 weeks of age) were obtained from the National Laboratory Animal Center (Taipei, Taiwan). All experimental procedures using these mice were performed in accordance with protocols approved by the Institutional Laboratory Animal Care and Use Committee of National Taiwan University. For the subcutaneous model (n=8), each mouse was inoculated s.c. in the dorsal flank with 2 × 10^6^ PLC5 cells suspended in 0.1 ml of serum-free medium containing 50% Matrigel (BD Biosciences, Bedford, MA). When tumors reached 100–200 mm^3^, mice received sorafenib (10 mg/kg) p.o. once daily. Tumors were measured twice weekly using calipers and their volumes calculated using the following standard formula: width × length × height × 0.523. The tumor samples were collected at the end of treatment for further investigation.

### Immunohistochemistry

Tumors from hepatocellular carcinoma patients who received surgical resection, post-operative treatment and follow-up in Changhua Christian Hospital from June 2012 to June 2013 were enrolled for analysis (CCH IRB No. 120504). Paraffin-embedded hepatocellular carcinoma tissue sections (4-μm) on poly-1-lysine-coated slides were deparaffinized and rinsed with 10 mMTris-HCl (pH 7.4) and 150 mM sodium chloride. Paroxidase was quenched with methanol and 3% hydrogen peroxide. Slides were then placed in 10 mM citrate buffer (pH 6.0) at 100°C for 20 minutes in a pressurized heating chamber. After incubation with 1:50 dilution of PKM2 antibody (Rabbit monoclonal to PKM2, Abcam, Cambridge, UK) for 1 hour at room temperature, slides were thoroughly washed three times with phosphate buffered saline. Bound antibodies were detected using the EnVision Detection Systems Peroxidase/DAB, Rabbit/Mouse kit (Dako, Glostrup, Denmark). The slides were then counterstained with hematoxylin. Negative controls had the primary antibody replaced by phosphate buffered saline. The expression of PKM2 was assessed semiquantitatively based on the intensity of staining and percentage of cell involved by a board certified pathologist. The intensity of staining was scored as negative, low and high. This study was approved by the ethics committee of the Institutional Review Board of Changhua Christian Hospital. All informed consents from sample donors were in accordance with the Declaration of Helsinki and were obtained at the time of their donation.

### Statistical analysis

Comparisons of mean values were performed using the independent samples *t* test in SPSS for Windows 11.5 software (SPSS, Chicago, IL).

## SUPPLEMENTARY FIGURES AND TABLE


