# Supplementary material for: SH2 domain-containing phosphatase 1 regulates pyruvate kinase M2 in hepatocellular carcinoma
Source: Oncotarget. 2016 Mar 5;7(16):22193–205. doi: 10.18632/oncotarget.7923 (PMC5008355; doi:10.18632/oncotarget.7923)
Supplement: Supplementary file 1 [file oncotarget-07-22193-s001.pdf]

SUPPLEMENTARY FIGURES AND TABLE

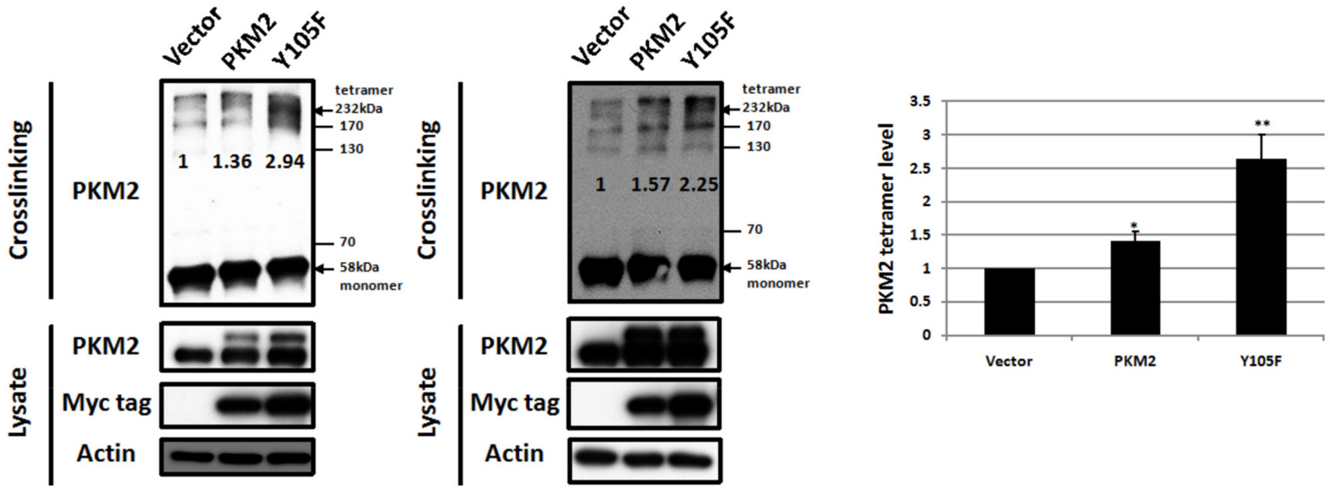

Supplementary Figure S1: The Y105F mutant of PKM2 increases the formation of tetrameric PKM2. PLC5 cells with wild-type or mutant PKM2 were crosslinked by 1% glutaradehtde before WB analysis.

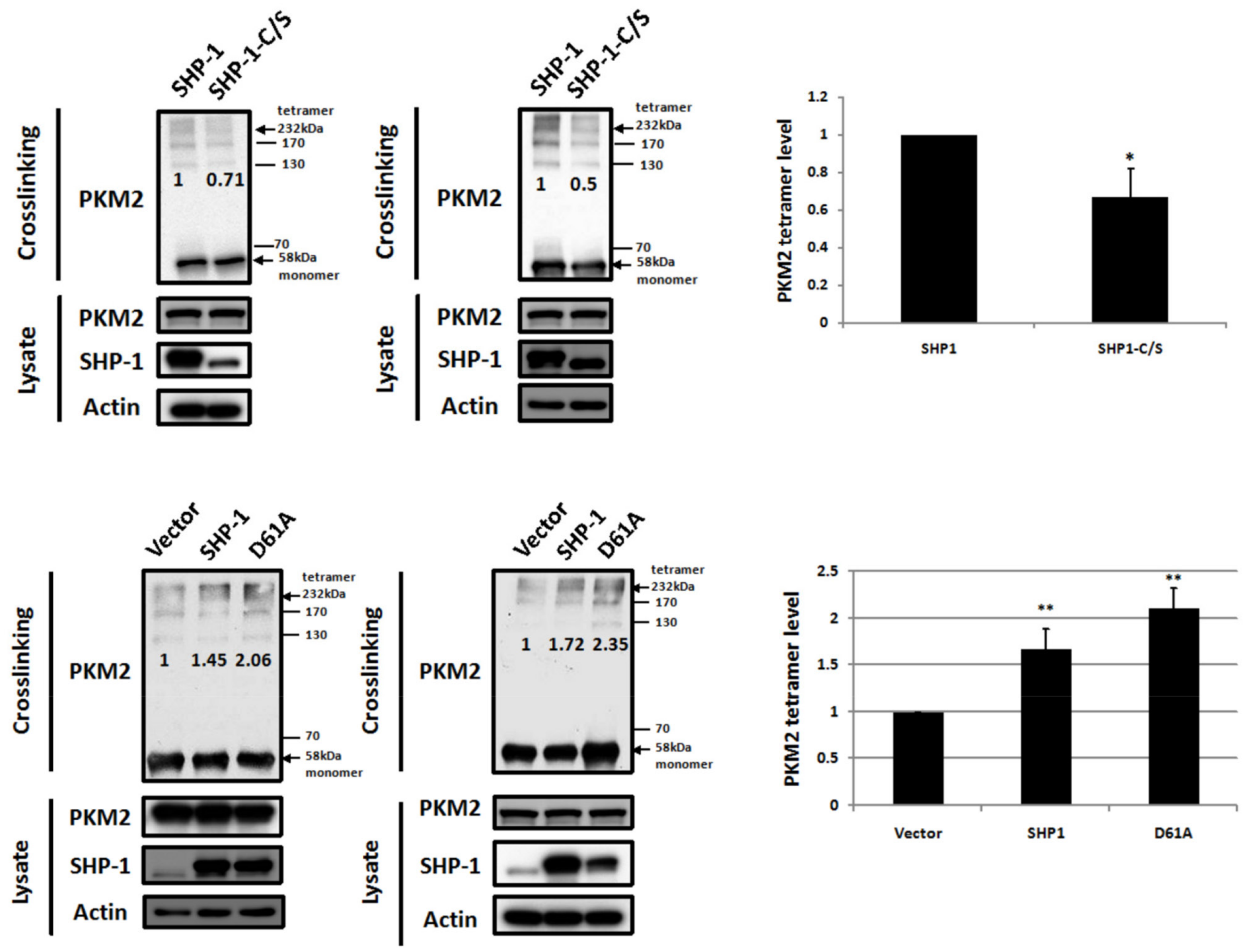

Supplementary Figure S2: The effect of SHP-1 on tetrameric PKM2. The catalytic-dead (C/S) and constitutively active mutant (D61A) of SHP-1 reduced and increased the tetramer formation of PKM2 respectively.

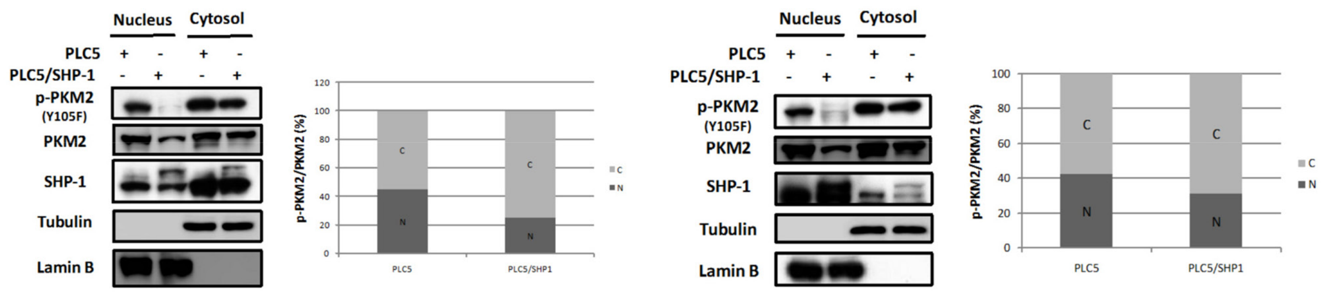

Supplementary Figure S3: SHP-1 decreases the nuclear percentage of p-PKM2.

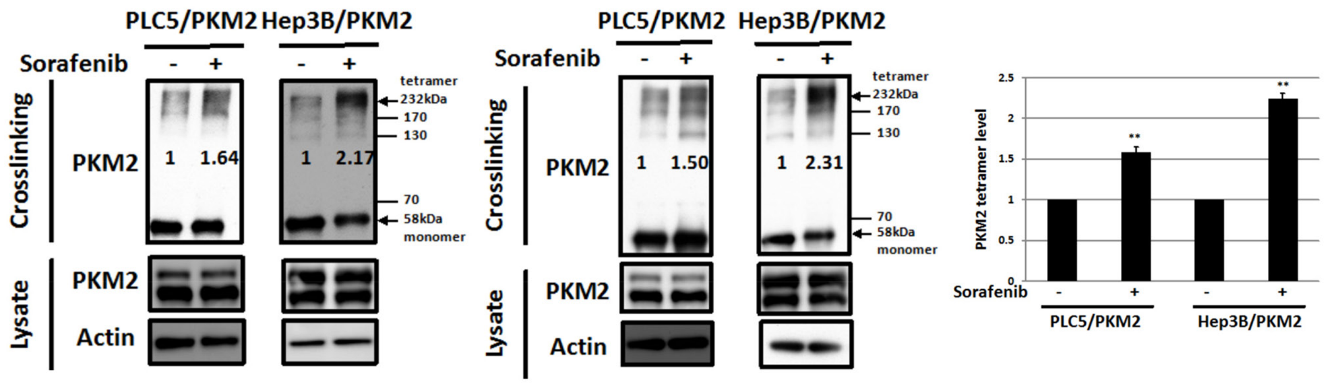

Supplementary Figure S4: Sorafenib increases the tetramer formation of PKM2.

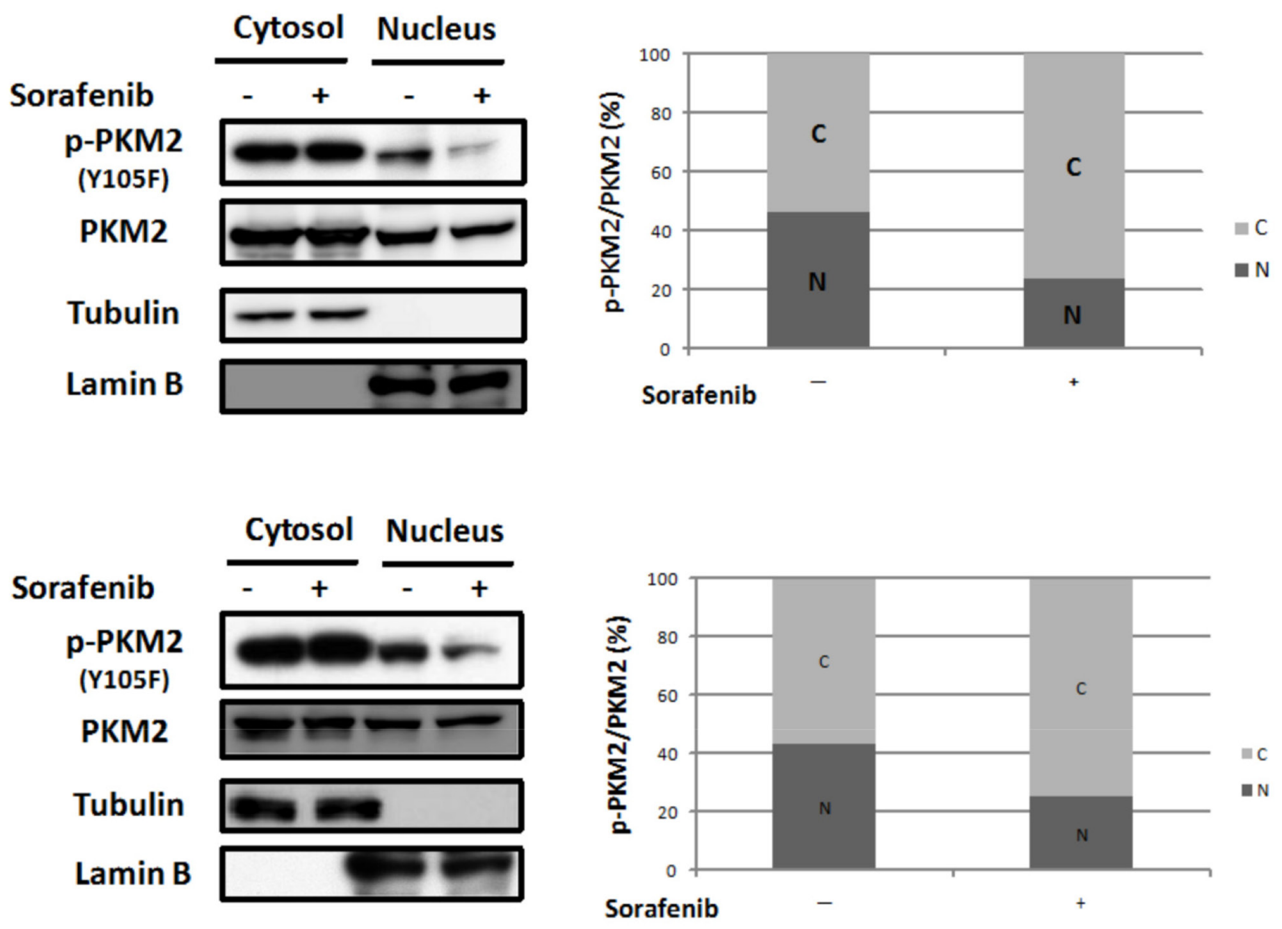

Supplementary Figure S5: Sorafenib reduces the nuclear fraction of p-PKM2.

Supplementary Table S1: General characteristics of patients (n=147)

Characteristics	N	%
Male gender	106	72.1
Median age (IQR)	63 (57- 72)	
Initial tumor number		
Single	123	33.3
2	13	55.1
multiple	11	11.6
Initial tumor size (cm)		
<3	72	49.0
3-5	36	24.5
>5	39	26.5
Positive nodal involvement	7	4.8
Positive PKM2 staining	59	40.1
Recurrence after primary surgical resection	26	17.7
< 6 month	15	10.2

Abbreviation: IQR, *interquartile range*.
